# Instrument-dependent variability affects both microplastic polymer identification and quantification: an inter-laboratory comparison using polymer standards

**DOI:** 10.1038/s41598-026-64477-w

**Published:** 2026-07-28

**Authors:** Martine Graf, David R. Chadwick, Yingming Sun, Fan Ding, Siyang Ren, Kai Wang, Changrong Yan, Shitong Li, Xuejun Liu, Mona Tolba, Ahmed Mosa, Sanjay Singh, Tapan Adhikari, Asit Mandal, Jyoti K. Thakur, Ajay Yadav, Meththa Gimhani, Achini Dias, Surani Chathurika, Mojith Ariyaratne, Hao T. T. Dang, Hien V. Nguyen, Tien T. Minh, Samantha Viljoen, Davey L. Jones

**Affiliations:** 1 School of Environmental & Natural Sciences, Bangor University, Bangor, Gwynedd LL57 2UW UK; 2 College of Land and Environment, Shenyang Agricultural University, Shenyang, China; 3 State Key Laboratory of Nutrient Use and Management, College of Resources and Environmental Sciences, Key Laboratory of Plant-Soil Interactions of Ministry of Education, National Academy of Agriculture Green Development, National Observation and Research Station of Agriculture Green Development (Quzhou, Hebei), China Agricultural University, Beijing, China; 4 Institute of Environment and Sustainable Development in Agriculture, Chinese Academy of Agricultural Sciences, Beijing, China; 5 Soils Department, Faculty of Agriculture, Mansoura University, Mansoura, Egypt; 6 Plant Nutrition Department, National Research Centre, Giza, Egypt; 7 ICAR – Indian Institute of Soil Science, Bhopal, India; 8 Sri Lanka Institute of Nanotechnology, Homogama, Sri Lanka; 9 Department of Urban Bioresources, Faculty of Urban and Aquatic Bioresources, University of Sri Jayawardenepura, Nugegoda, Sri Lanka; 10 Department of Crop Science, Faculty of Agriculture, University of Peradeniya, Peradeniya, Sri Lanka; 11 Soils and Fertilizers Institute, Ha Noi, Vietnam; 12 Bioplastic Innovation Hub, Food Futures Institute, Murdoch University, Perth, Australia

**Keywords:** ILC, Vibrational spectroscopy, Analytical uncertainty, Reference libraries, Interlaboratory validation, Method standardisation, Chemistry, Environmental sciences

## Abstract

Microplastic analysis is a critical tool for environmental risk assessments and policy development, aligning with several UN Sustainability Goals, however the extent to which analytical outcomes depend on instrument type and spectral reference libraries remains poorly quantified. Here, we present an inter-laboratory comparison study across seven countries evaluating both infrared polymer identification and fluorescent microscopy quantification, using pristine individual polymer standards, mixed polymer standards, and controlled recovery experiments. Participants used standardised protocols and reference materials, differing only in instrument manufacturer, type, reference library, and observer. Our results demonstrate substantial inter-laboratory variability in spectra match quality, polymer identification accuracy, polymer composition in mixed samples, and recovery rates from standardised matrices. Our findings highlight strong instrument- and library-dependent effects on data output despite the implementation of standardised protocols. Collectively, our findings demonstrate that protocol standardisation alone is insufficient to ensure inter-laboratory comparability and that substantial uncertainty persists in reported microplastic composition and abundance. We therefore propose a collaborative approach between researchers and instrument manufacturers encompassing library harmonisation, instrument calibration, and transparent reporting. Without such measures, reported microplastic composition and abundance may continue to reflect laboratory-specific artefacts rather than true environmental variability, ultimately limiting the confidence and policy impact of the reported results.

**Supplementary Information:**

The online version contains supplementary material available at https://doi.org/10.1038/s41598-026-64477-w.

## Introduction

Microplastics (MPs) are recognised as persistent anthropogenic contaminants across all environmental matrices^1–4^. Their reported abundance, composition, and size distribution are used globally to inform risk assessments, regulatory frameworks, and mitigation strategies, highlighting the importance of data comparability and reliability. However, MP analysis from environmental matrices is still an evolving field, usually involving a multi-stage process of sampling, extraction, quantification, and identification, which can rely heavily on instrument types and software-based interpretations, introducing uncertainty^5–11^.

Infrared (IR) spectroscopy, including but not limited to Fourier-transform infrared (FTIR) and Raman, has been established as a widely used method for polymer identification, due to its accessibility, processing speed, and cost effectiveness^12,13^. Polymer identification via IR spectroscopy operates by matching particle spectra against library spectra of known polymers, often using match quality scores as an indicator for accuracy (i.e. the higher the match quality, the better the match, the more reliable the identification). However, these reference libraries are mostly manufacturer/instrument-specific and differ in composition, spectral pre-processing, and matching algorithms within the respective instrument-specific software^5,13,14^. The extent to which these factors influence outputs, however, remains insufficiently quantified, creating uncertainty regarding accuracy and comparability of reports.

Along the same lines, fluorescent microscopy is a widely applied technique for MP particle quantification, due to its ease of use and cost-effectiveness^15–18^. Whilst it has been acknowledged that this method holds a level of uncertainty strongly linked with sample preparation, as fluorescent dye may not only adhere to polymer particles but also organic matter, it is a simple method for processing large quantities of samples. However, whilst there is broad consensus within the research community calling for the implementation of standardised extraction and sample preparation procedures^19,20^, individual instruments still differ in light sources, detector sensitivity and minimum resolvable particle size. Further variability may also arise from differences in chemical suppliers and image processing parameters across laboratories. These factors may introduce systematic biases in particle quantification, potentially leading to over- or underestimation of recovery rates and environmental MP concentrations.

Whilst progress towards standardisation has been made through prior inter-laboratory comparison studies (ILCs)^6–11^, uncertainty remains regarding the influence of polymer types, analytical configuration, and reference library composition when laboratories follow harmonised protocols. Previous ILCs have substantially improved understanding of analytical reproducibility and have highlighted the need for protocol harmonisation, particularly with respect to extraction performance, particle recovery, and the analysis of complex environmental or reference matrices. However, comparatively little attention has been given to polymer-specific identification performance, the relationship between spectral match quality and identification accuracy, the influence of reference library composition, or how these factors propagate into reported polymer composition and quantification outcomes. Additionally, although previous studies have independently assessed spectroscopic identification and particle recovery, the combined influence of infrared polymer identification and fluorescence-based quantification under harmonised analytical protocols has not, to our knowledge, been systematically evaluated within a single international comparison study. Understanding these additional sources of analytical uncertainty is essential to determine whether reported differences in microplastic abundance and polymer composition reflect genuine environmental variability or analytical artefacts.

To address these knowledge gaps, we conducted an international inter-laboratory comparison involving seven laboratories to systematically evaluate the influence of polymer type, analytical configuration, and reference library on both IR-based polymer identification and fluorescence-based quantification following implementation of a standardised protocol. Using pristine individual polymer standards of 9 conventional polymers, controlled polymer mix standards, and recovery experiments in standardised matrices, we assessed (i) spectral match quality distributions; (ii) polymer identification accuracy; (iii) compositional bias in mixed samples; and (iv) recovery rate variability from standard matrices. In addition, we investigated the influence of reference library composition through a controlled library-modification case study using a single instrument. Together, these experiments were designed to determine how analytical configuration influences polymer identification and quantification and to provide evidence-based recommendations for improving the reliability and comparability of environmental microplastic analyses.

## Results

### Polymer difficulty drives accuracy predictive strength of match quality scores

Whilst the median match quality across countries ranged from 85.2% − 98.1%, the overall accuracy (i.e. correctness of identification) ranged from 54.4% (Egypt) to 93.3% (UK) (Table 1). This shows a substantial variability in identification performance between laboratories. The median match quality scores for incorrectly identified particles were on average lower than for correctly identified particles, except for Egypt, where median match quality was almost identical (Table 2), suggesting a systematic overconfidence in quality rather than random misclassification. Additionally, China showed good accuracy (88.9%) but the overall lowest median match quality (Table 1), indicating that (i) match quality does not automatically reflect correctness, and (ii) that match quality thresholds are not comparable across laboratories and need to be evaluated on an individual basis. This is supported by match quality thresholds (70–95%) performing differently for each country (Table 3). At a 90% threshold setting, some countries retained large proportions of correct identifications (e.g. India, Australia), whilst others excluded a substantial number of correct matches (e.g. China, UK, Vietnam). In Egypt, a large proportion of incorrect identifications exceeded the 90% threshold, indicating that thresholding match quality alone would not resolve the issue of consistent misclassification (Table 3). Overall, results show that optimal match quality thresholds vary substantially between countries, suggesting match quality thresholds are laboratory-specific and should not be applied universally across IR datasets.

**Table 1:** Accuracy and median match quality of individual polymer standards by laboratories located in different countries (*n* = 90; 9 polymer types x 10 particles each). India was unable to analyse the PTFE standard (therefore *n* = 80; 8 polymer types x 10 particles each). Data are expressed as mean (accuracy) and median (match quality).

Country	*n*	Accuracy (%)	Median match quality (%)
Australia	90	88.9	96.2
China	90	88.9	85.2
Egypt	90	54.4	97.0
India	80	87.5	98.1
UK	90	93.3	91.8
Vietnam	90	77.8	91.1

**Table 2:** Median spectral match quality of correctly (true) and incorrectly (false) identified standard polymer particles, grouped by laboratory in each country. *n* = number of particle identifications in each category.

Country	Correctness	*n*	Median match quality (%)
Australia	False	10	89.5
	True	80	96.3
China	False	10	77.1
	True	80	86.2
Egypt	False	41	97.0
	True	49	96.0
India	False	10	81.7
	True	70	99.3
UK	False	6	85.9
	True	84	93.1
Vietnam	False	20	88.8
	True	70	91.4

**Table 3:** Percentage of polymer particles incorrectly and correctly identified when applying spectral match quality thresholds ranging from 70 to 95%, for laboratories in each participating country.

Country	*n*	% of incorrectly identified polymers with match quality ≥				% of correctly identified polymers with match quality ≥			
		70%	80%	90%	95%	70%	80%	90%	95%
Australia	90	11	11	2	1	89	80	73	60
China	90	11	2	0	0	84	66	31	17
Egypt	90	44	44	44	44	52	47	33	28
India	80	13	13	2	0	86	88	86	74
UK	90	7	7	1	0	92	91	63	37
Vietnam	90	22	14	11	2	78	78	51	11

**Fig. 1:**
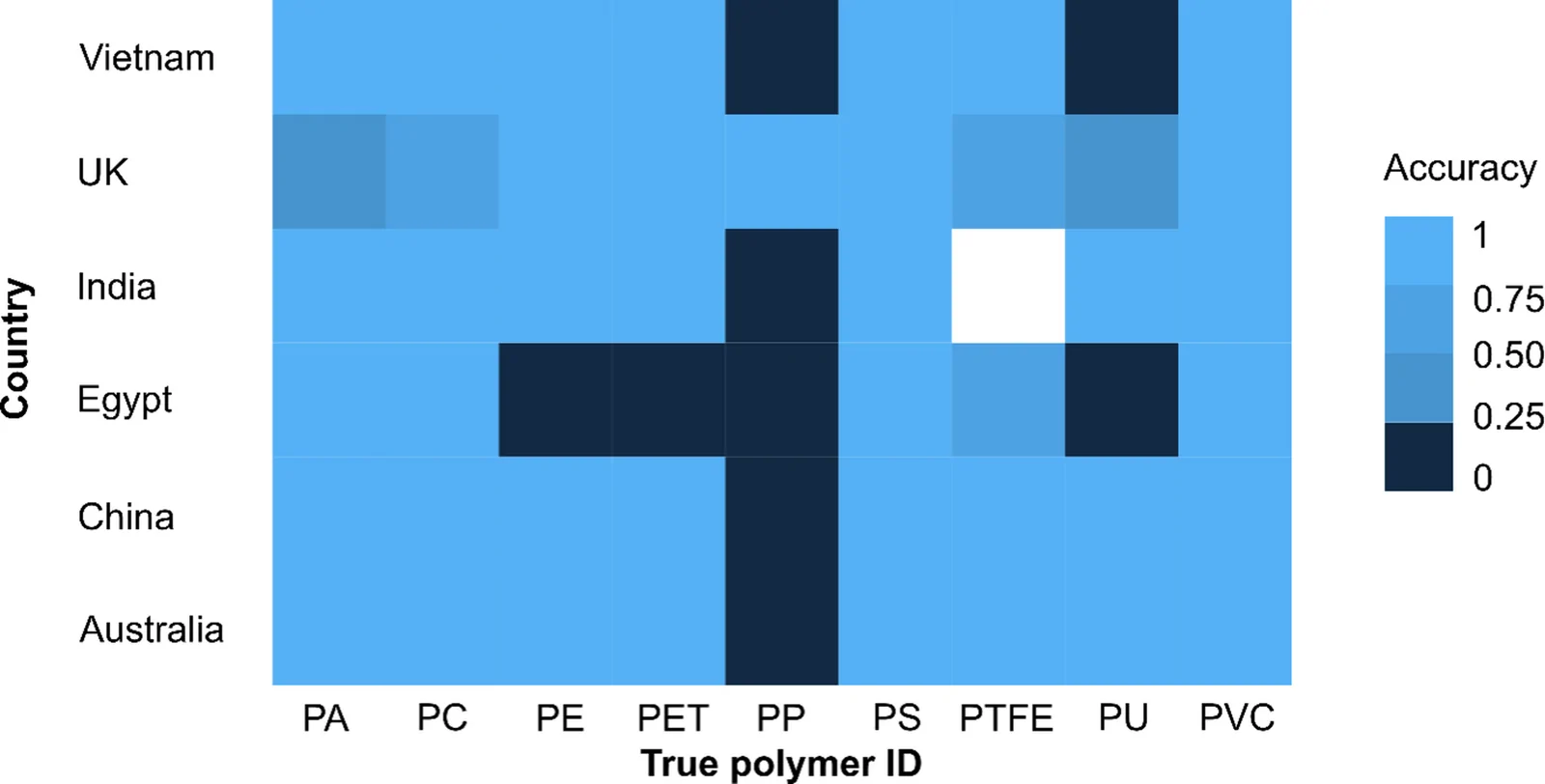
Heatmap displaying the accuracy of identification for each individual polymer standard by laboratories located in different countries (*n* = 10 particles per polymer type). PTFE was not analysed in India, and is therefore shown in white in the heatmap. The colour scale ranges from 0 to 1, where 1 indicates perfect identification accuracy.

**Fig. 2:**
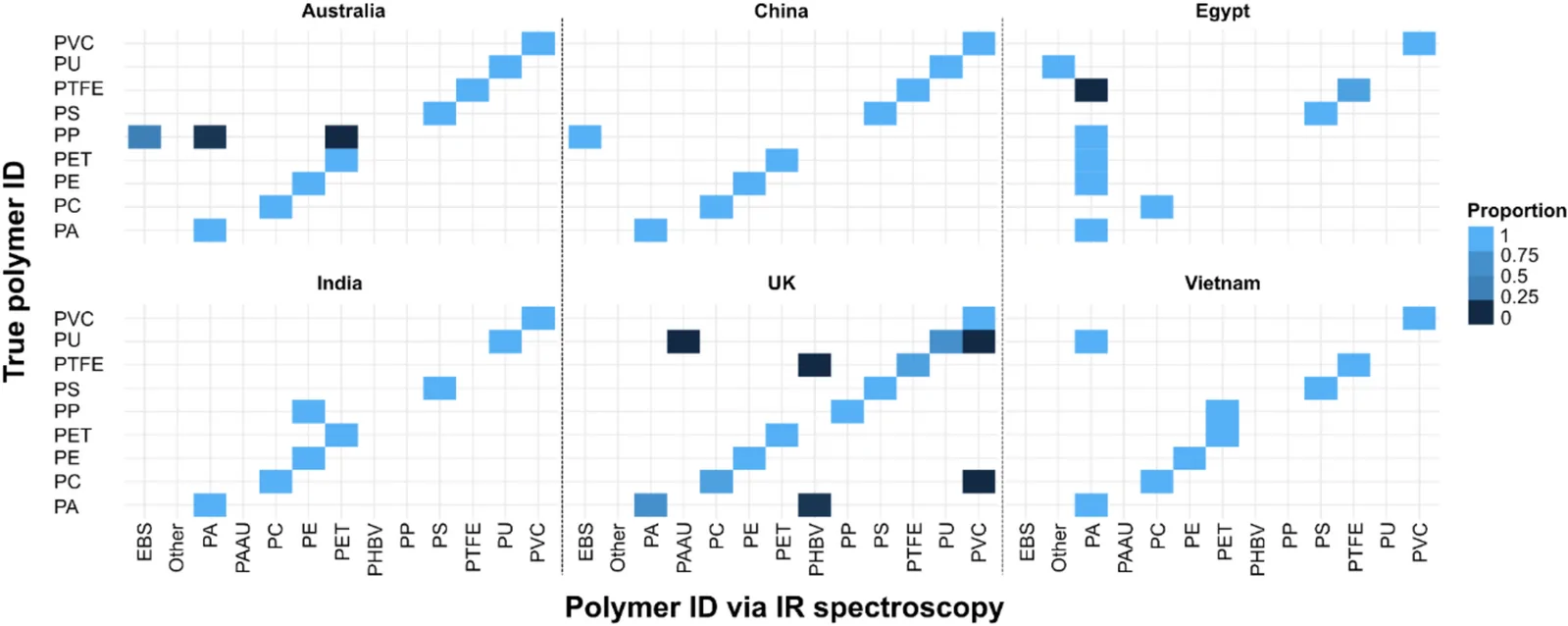
Confusion matrices for individual polymer standards by laboratories located in different countries. Each panel represents one country. Rows indicate the true polymer identity of the standard, and columns indicate the best library match. Tile colour intensity represents the proportion of particles (from *n* = 10) assigned to each identity. ‘Other’ represents non-polymer identifications.

**Fig. 3:**
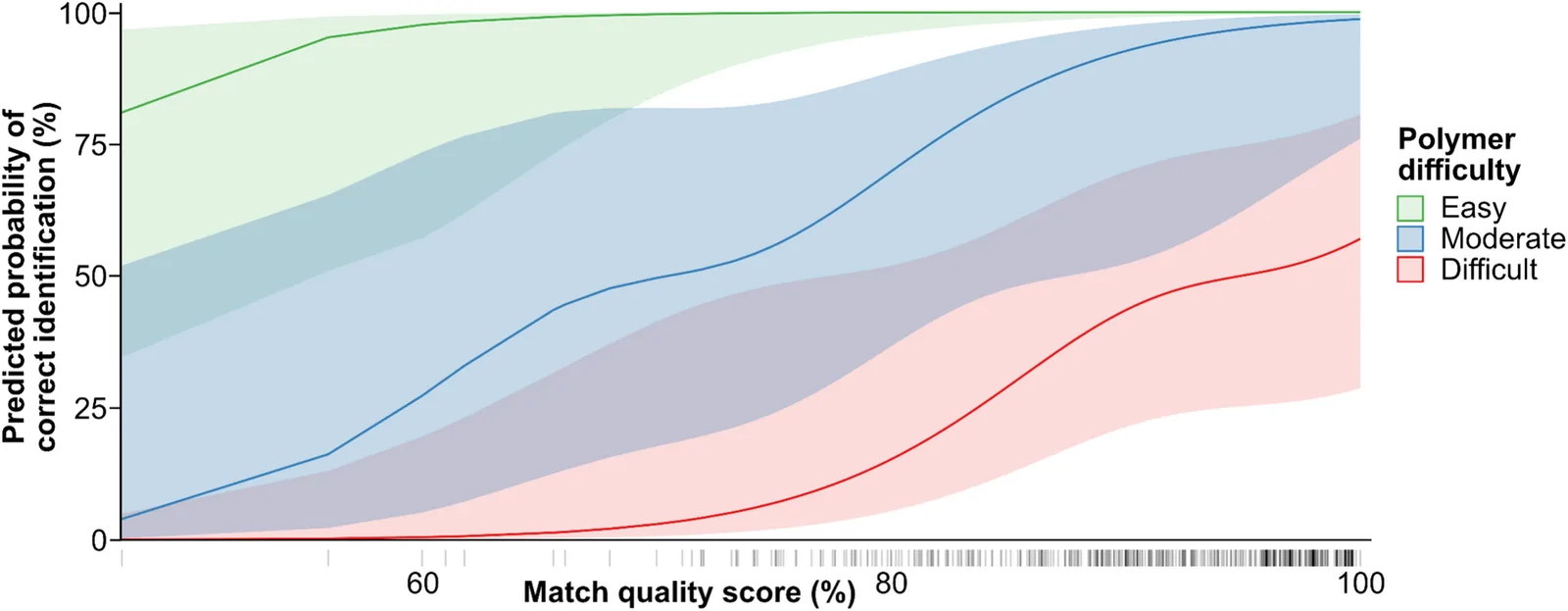
Predicted probability of correct polymer identification (%) as a function of match quality score (%), with polymers grouped by identification difficulty (easy: PS, PVC, PTFE; moderate: PET, PC, PA, PE; difficult: PP, PU). Lines represent population-level predicted probabilities accounting for inter-laboratory variability. Shaded areas represent 95% confidence intervals. Rug marks along the x-axis show the distribution of observed match quality scores.

Polymer-specific accuracy also varied greatly across countries. Whilst certain polymer types were consistently well identified across countries (e.g. PVC, PS), others were much more variable (e.g. PP, PU) (Fig. 1). Particularly PP was misidentified in almost all countries (Fig. 2). This is supported by the GLMM, which showed a strong influence of polymer type on identification accuracy. As mentioned in the method section, models including all individual polymer identities showed signs of quasi-complete separation for polymers with near-perfect accuracy (e.g. PS, PVC), leading to unstable coefficient estimates, resulting in the grouping of polymers according to difficulty. Compared to easily identified polymers (PS, PVC, PTFE), the probability of accurate identification was significantly reduced for moderate polymers (PET, PC, PA, PE) (*β* = −6.54, *p* < 0.001), and reduced even further for difficult polymers (PP, PU) (*β* = −10.91, *p* < 0.001). The model also showed a significant positive association between match quality and accuracy (*β* = 1.44 per 1 SD increase, *p* < 0.001), although large inter-laboratory variability remained (SD = 2.37 on the log-odds scale), i.e. the strength of this relationship differed between laboratories. These results show that polymer difficulty dominates identification accuracy, whilst match quality scores have predictive value but are not universally applicable (Fig. 3). While match quality is strongly associated with accuracy for polymers of moderate difficulty, easy polymers show high accuracy even at low match quality scores (< 60%), and difficult polymers remain frequently misidentified even at high match quality (> 95%), with a predicted probability of correct identification just above 50% (Fig. 3).

### Library composition as key factor for accuracy and match quality

**Fig. 4:**
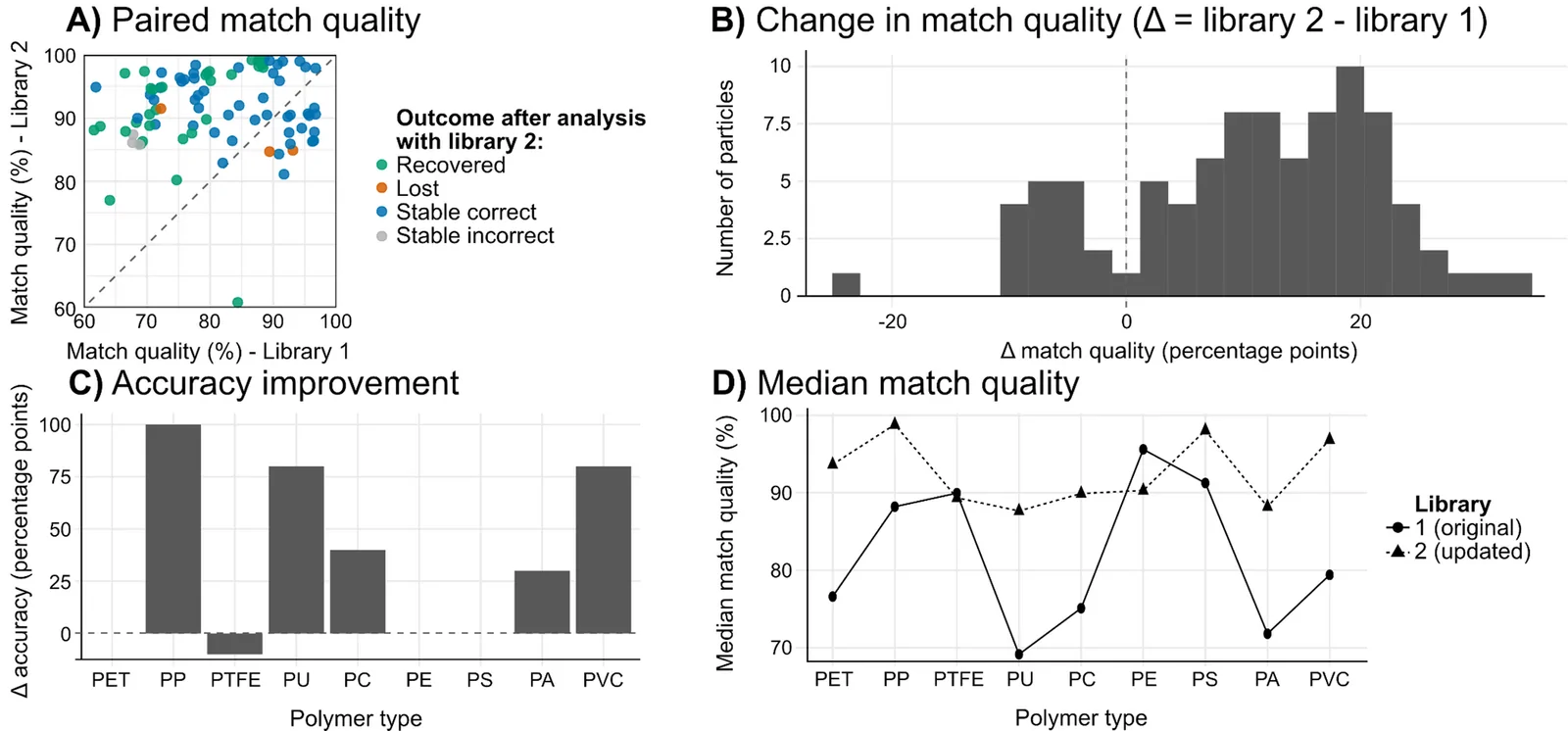
Effect of reference library on match quality and identification accuracy for UK polymer standards analysed using LDIR and two different spectroscopic libraries (*n* = 90, 9 polymer standards x 10 particles each). **(a)** Paired match quality scores for the same particles analysed using the original (library 1) and updated (library 2) libraries. Points are coloured by outcome: recovered (incorrect with library 1, correct with library 2), lost (correct with library 1, incorrect with library 2), stable correct (correct with both libraries), stable incorrect (incorrect with both libraries). **(b)** Distribution of changes in match quality (Δ = library 2 − library 1). **(c)** Change in identification accuracy (percentage points) per polymer standard following library modification. **(d)** Median match quality per polymer for each library, illustrating polymer-specific changes.

Updating the reference library with the spectra of standard polymers used in this study resulted in a substantial improvement in identification accuracy and median match quality. Median match quality across all particles significantly increased from 80.4% (library 1 = original library) to 91.8% (library 2 = updated library), representing an 11.4% point increase (*p* < 0.0001). Similarly, the average accuracy across all standard particles (*n* = 90) increased from 58% to 93%, corresponding to an improvement in accurate identification of previously incorrect particles of 92.1% (equivalent to 35 out of 38 particles) (Fig. 4a). Only 3 out of 52 correctly identified particles from library 1 were misidentified with library 2, corresponding to an introduced error rate of 5.8% (Fig. 4a). For particles whose identification was correct under library 1, median match quality increased by an average of 15.8% points (Fig. 4b). Improvements were also polymer-specific, with the largest increases in accuracy observed for PP, PU, and PVC, and no changes to polymers which were already consistently accurately identified with library 1 (PET, PE, PS) (Fig. 4c). However, improvements in accuracy were not directly related to improvements in median match quality for individual polymer types, e.g. PET showed no change in accuracy but a large increase in median match quality (Fig. 4d). It should be noted that the updated library was evaluated using the same polymer standards from which the additional spectra were derived, therefore, the magnitude of the observed improvement should not be interpreted as an independent estimate of library performance.

### Distribution patterns of polymer types in mixes reflect polymer-specific difficulty and laboratory-specific analytical performance

**Fig. 5:**
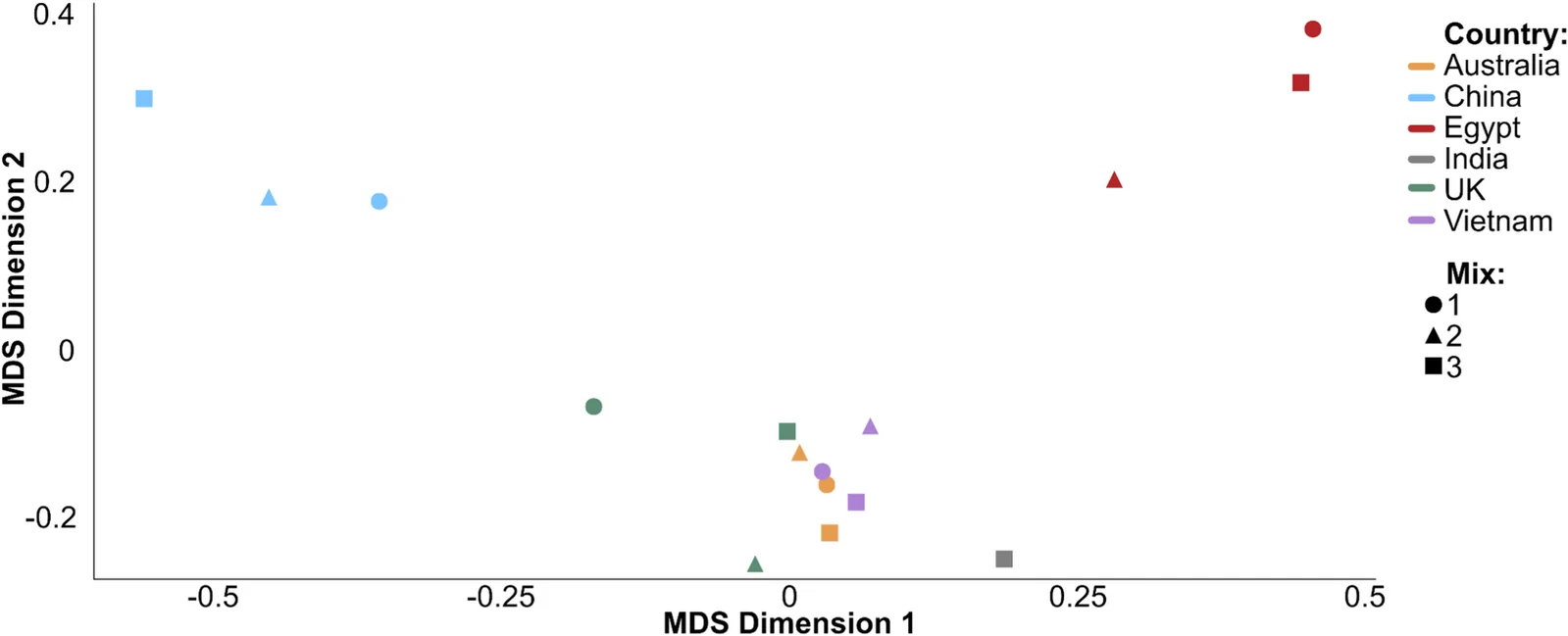
Multidimensional scaling (MDS) ordination of mixed-polymer standard compositions across countries. Points represent individual mix replicates (1–3, with *n* = 50 each) for laboratories in each country based on compositional similarity in polymer identification. Distances between points reflect differences in polymer distributions.

Reported polymer composition in the provided mix (*n* = 3) differed between countries, despite identical source material. For the majority of countries, easy and moderate polymers dominated the identification, with only a low proportion of difficult polymers (Table 4). The consistency between triplicate mixes within a country was generally higher than the similarity between countries, indicating that intra-laboratory workflows were internally consistent but differed between countries (Fig. 5). Countries with low accuracy for difficult polymers in the individual standards, showed a tendency to underreport difficult polymers in mixes, instead showing bias toward moderate and easy polymers. The results show that the reported mix composition does not reflect the true mix composition (Table S7), instead reflecting polymer-specific difficulty and inter-laboratory differences in analytical performance.

**Table 4:** Proportion of polymer difficulty classes reported in polymer standard mixes (*n* = 50 particles per mix; ‘easy’ = PTFE, PS, PVC; ‘moderate’ = PET, PC, PA, PE; ‘difficult’ = PP, PU)). Not shown are incorrect identifications of polymers that were not included in the mix (i.e. identifications that were not any of the 9 polymers present in the mix). Mix 1–3 refers to replicate 1–3 of the same mix composition.

Country	Mix	Proportion of “difficult” polymers (%)	Proportion of “moderate” polymers (%)	Proportion of “easy” polymers (%)
Australia	1	16	54	28
	2	14	48	38
	3	14	70	16
China	1	2	48	50
	2	2	32	66
	3	0	24	76
Egypt	1	2	24	8
	2	0	34	26
	3	0	20	20
India	3	48	14	36
UK	1	4	50	46
	2	8	64	28
	3	16	18	64
Vietnam	1	6	52	34
	2	0	64	20
	3	46	16	38

To explore potential physical drivers of polymer identification difficulty, median particle size, particle type, and colour (Table S6) were examined at polymer level. Polymer difficulty could not be explained by particle size alone, as difficult polymers spanned more than two orders of magnitude in median particle size and included both the smallest (PP) and largest (PU) particles (Fig. S1). There was no observed relationship between polymer colour and difficulty. Whilst difficult polymers were disproportionately represented among powder particles, it is noteworthy that the sample size (*n* = 9 polymer types) is limited, therefore these observations should be treated as informative, not definitive (i.e. association not causation). It is also important to note that the influence of particle size on polymer identification is unlikely to be linear. Very small particles may produce spectra with lower signal-to-noise ratios or be more susceptible to optical limitations, whereas very large or irregular particles may introduce different spectral artefacts associated with particle morphology, focal depth, or sampling geometry depending on the analytical platform. Consequently, particle size alone is unlikely to explain identification performance, and future studies should evaluate particle size, morphology, and polymer identity independently using dedicated reference materials spanning the operational size range of different spectroscopic techniques.

### Quantification of MPs in solid matrices is highly variable and instrument-dependent

**Fig. 6:**
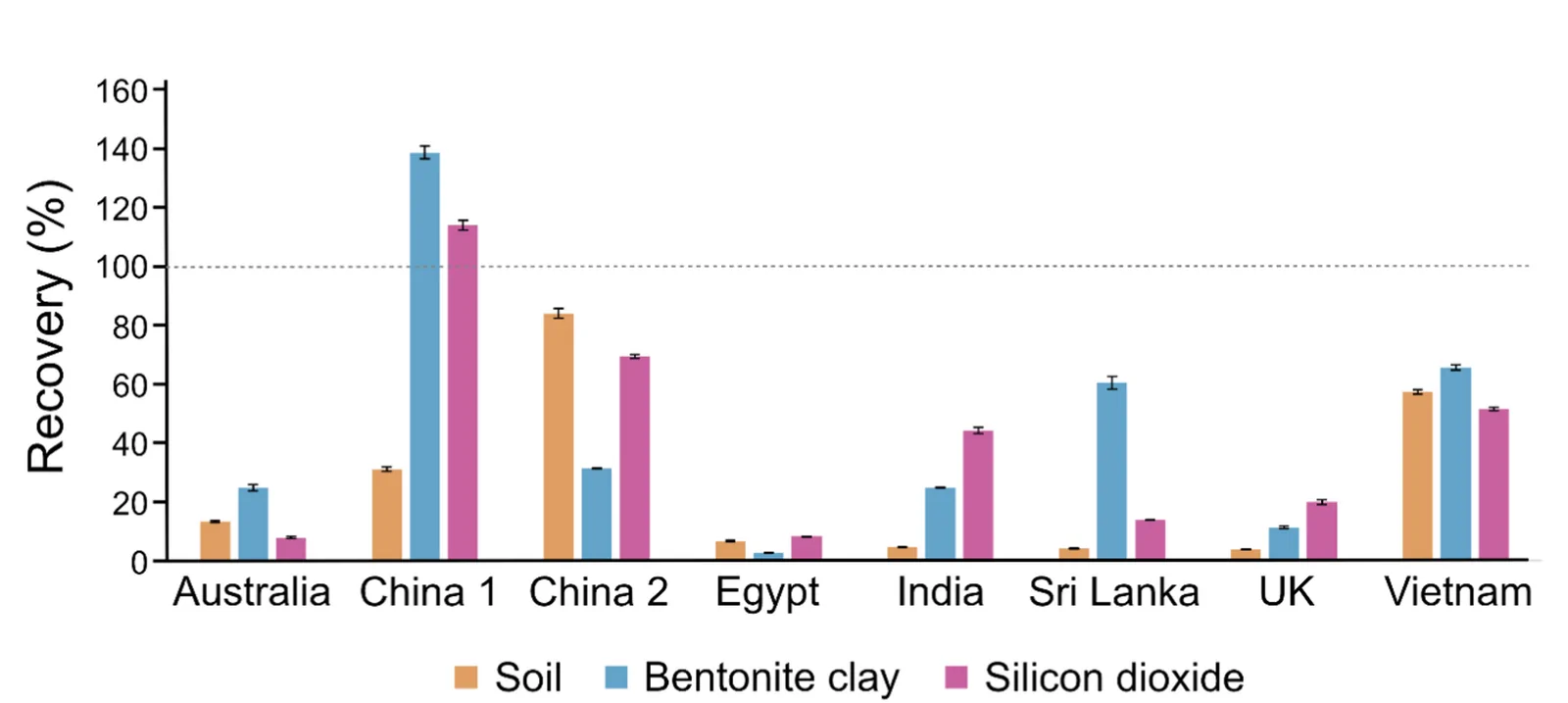
Recovery rates of PE particles (0.04% *w*/*w*) from bentonite clay, silicon dioxide (quartz sand), and country-specific soil matrices after application of a standardised extraction method. Recovery rates were calculated against the estimated PE particle number added to each sample. Data are presented as mean ± SE (*n* = 3). Countries correspond to one participating laboratory per country, except for China, where two laboratories participated (differentiated numerically).

The recovery of PE from spiked matrices (silicon dioxide, bentonite clay, country-specific soil) varied substantially between laboratories despite the use of harmonised protocols. The average recovery rate ranged from < 5% to > 130% depending on laboratory and matrix (Fig. 6). Whilst high variability in soil recovery rates was expected due to differences in soil types, the recovery rates were overall lower than anticipated with < 80% for 7 out of 8 laboratories (Fig. 6). In contrast, recovery rates for bentonite clay and silicon dioxide matrices were expected to be similar across laboratories, but showed high inter-laboratory variation as well (Fig. 6). Notably, China 1 reported recovery rates exceeding 100% for bentonite clay and silicon dioxide, indicating a systematic particle overestimation, whereas all other laboratories consistently underestimated particle numbers (Fig. 6). Despite blank-correction, normalisation of particle counts to particles g^− 1^ dry weight, and ImageJ threshold optimisation per laboratory, variability persisted, suggesting that fluorescence imaging characteristics contributed strongly to inter-laboratory differences.

## Discussion

### Match quality thresholds are not universal, and accuracy prediction is strongly polymer dependent

The findings from the individual standard comparison demonstrate that IR polymer identification accuracy varies substantially between laboratory-specific analytical configurations, even when analysing identical materials (Table 1; Fig. 1). This aligns with findings from previous inter-laboratory comparisons reporting polymer identification accuracy between 53% − 100% using µFTIR/ATR-FTIR^8^. Crucially, spectral match quality values were not directly comparable between laboratories in our study. The median match quality for incorrectly identified particles in Egypt exceeded the median match quality for correctly identified particles in four other countries (Table 2), indicating that match quality scales are instrument- and library-specific. This finding is particularly surprising given that Egypt and India both used the same open-source reference library (Open Specy^5^ yet achieved identification accuracies of 54.4% and 87.5% respectively, suggesting that differences in analytical processing prior to library matching contribute substantially to the observed inter-laboratory variability, independent of the shared reference library. Similarly, whilst Australia and Vietnam used the same instrument and manufacturer supplied reference library, accuracy and match quality scores still differed, highlighting the importance of polymer-specific validation.

The varied retention of incorrect and correct polymer identifications at different match quality thresholding levels (Table 3), provides further evidence that universal match quality thresholds are inappropriate and that instrument-specific calibration is required for polymers of interest. Additionally, results showed that the reliability of match quality as an indicator for accuracy is highly polymer dependent (Fig. 2). Thresholding at commonly applied levels of 70–80%^20^ would retain easy polymers with > 95% accuracy, whilst accuracy of moderate polymers decreases to just above 50%, and below 20% for difficult polymers (Fig. 3). This highlights that certain polymers are intrinsically more sensitive to spectral library representation and matching algorithm performance. Inter-laboratory comparisons must therefore account for both polymer identity and reference library configuration, emphasising the need for polymer-specific QA/QC procedure implementation into analytical protocols, as well as transparency and harmonisation of libraries across instruments and reporting of workflows^5,13^.

To facilitate implementation, we propose a simple validation framework for laboratories (Fig. S2). First, laboratories should analyse a selection of known polymer standards, including common polymers that are reported across many studies (e.g. PP, PE, PVC) as well as potentially more rare polymers that laboratories suspect to find in their study. The inclusion of common polymer standards will allow comparability between laboratories/matrices, and as shown here, “easy” polymers can act as internal performance benchmarks, whereas “difficult” and “moderate” polymers provide a sensitivity test of the laboratory’s ability to distinguish between different polymer types. Second, the relationship between each polymer’s match quality and identification accuracy should be established under the laboratory’s specific conditions (e.g. instrument, software, spectral reference library, pre-processing workflows). Third, polymer-specific match quality thresholds should be selected based on the best balance between false-positive and false-negative identifications. Lastly, thresholds should be tested using a mixed-polymer reference sample of known composition (e.g. equal particle numbers, equal weight) with and without sample matrix (as matrix has been shown to also influence match quality thresholds (Davis et al., under review)), before being applied to environmental samples. This approach would allow laboratories to establish reproducible QA/QC procedures while acknowledging that threshold values are not directly transferrable between analytical set-ups, matrices, and polymer types. Note, that this framework should be adjusted to include environmentally weathered reference polymers in the future to align better with expected match quality values of weathered versus pristine spectra.

### Instruments and libraries are key drivers for polymer identification, match quality, and accuracy

The results of the UK case study demonstrate that reference library composition plays a significant role in IR polymer identification accuracy (Fig. 4). The improvement in both match quality and accuracy following library modification with known polymer spectra indicates that previous misidentifications under library 1 can be attributed to incomplete or suboptimal spectral representation rather than intrinsic spectral ambiguity. Importantly, improvements were not achieved at the cost of introducing new misidentifications. The correct identification of approx. 92% of previously misidentified particles, combined with only approx. 6% newly misidentified particles, which were previously correct, provides strong evidence that library refinement for specific target polymer types enhances both confidence and reliability of outputs. Furthermore, the observed polymer-specific improvements highlight the variable sensitivity of polymer types to library structure, and their representation in libraries provided by manufacturers. This suggests that differences in accuracy and match quality may reflect variations in library composition as much as differences in instrument hardware, raising concern regarding the comparability of data generated under different laboratory-specific analytical configurations, or even the same configuration using different libraries.

An important limitation of this case study is that the updated reference library was generated using spectra from the same polymer standards that were subsequently re-analysed. Consequently, whilst the observed improvement clearly demonstrates that reference library composition can substantially influence polymer identification performance, the magnitude of the improvement should not be interpreted as an independent validation of library refinement and may represent an upper estimate of the benefits achievable under routine applications. Independent validation using separate particles of the same polymers, and ideally environmentally weathered reference materials, would be required to determine how well these improvements transfer to unknown environmental samples. Nevertheless, the findings demonstrate that incomplete spectral representation within a library can be a major source of polymer misidentification and highlight the importance of validating any library modifications prior to routine implementation. It is noteworthy that LDIR spectroscopy may be particularly susceptible to misidentification because it operates in a narrower spectral range (975–1800 cm^− 1^) than full FTIR spectroscopy, potentially missing some of the diagnostic bands that would distinguish polymers.

These findings provide strong evidence for the need for harmonised reference libraries for applications requiring inter-laboratory comparability, or at minimum consistent reporting of library composition, to enable meaningful cross-laboratory comparison of IR spectroscopy data. Whilst some open-source options for IR spectral libraries exist (e.g. Open Specy^5^, siMPle^14^, it is noteworthy that Egypt and India recorded spectra on different instruments, but exported and analysed them for identification using the same pre-processing workflows and the same library resource (Open Specy^5^. Despite this library harmonisation, polymer identification accuracy varied from 54.4% to 87.5%, strongly indicating instrument-specific effects. This highlights that whilst open-source libraries are a valuable resource, without the alignment of instrument-specific processing workflows that occur prior to spectra export, true comparability between laboratories is unlikely to be achieved.

It is also noteworthy that the implications of these findings differ depending on the analytical objective. For targeted studies where likely polymer types are known, such as source appointments or investigation of specific plastic products, supplementing libraries with representative spectra of known source materials may improve identification accuracy. However, any refined library should subsequently be validated using an independent set of reference materials that were not used during library development before being applied routinely to unknown samples. In these applications, maximising identification accuracy for the polymers of interest may be more important than maintaining strict comparability with other studies. However, for blind environmental surveys where target polymers are unknown and potentially rare, highly customised libraries may inadvertently bias identifications towards more common polymers. In contrast, broad-scale environmental monitoring programmes benefit from harmonised reference libraries, as consistent analytical workflows improve comparability between laboratories and over time, even though analytical artefacts remain. In this context, laboratories should use broad, transparent, and preferably harmonised reference libraries, apply polymer-specific validation using known reference materials, and avoid forced polymer assignments where spectra are ambiguous. We therefore recommend that uncertain identifications are reported using confidence categories or retained as unknown/other where appropriate, rather than being accepted solely on the basis of match quality.

### Mixed-sample analysis requires polymer-specific validation

The high variability in reported polymer composition from the standardised mix across countries demonstrates that inter-laboratory differences extend beyond the accuracy of individual polymer types and influence the compositional profile of mixed polymer samples. These differences further emphasise the influence of spectral library compositions and matching algorithms, as well as a likely particle selection bias under manual analysis. The overall consistency across triplicates within each country suggests that observed differences are systematic by country and not random artefacts (Fig. 5), providing strong evidence that laboratory-specific analytical configurations systematically influence reported mixed-sample composition. It should be noted that India analysed only a single replicate owing to sample loss. Consequently, within-laboratory repeatability could not be assessed for India, although this does not affect the broader comparison of polymer composition between laboratories. The high repeatability observed within individual laboratories has important implications for environmental monitoring programmes. The close agreement among replicate analyses suggests that laboratory-specific workflows are internally consistent, supporting the detection of temporal trends when the same analytical configuration is maintained throughout a monitoring programme. However, the substantial differences observed between laboratories indicate that comparisons across monitoring programmes, or even within long-term programmes following changes in instrument, software, or reference library, should be interpreted with caution. Where multiple laboratories contribute to monitoring networks, the routine use of shared reference materials and periodic inter-laboratory calibration exercises would help distinguish analytical variability from genuine environmental change.

Furthermore, polymers identified as ‘difficult’ in the individual standard analysis showed a general underrepresentation in the majority of countries, emphasising the link between polymer-type accuracy and mixed-sample outcomes. We have shown that IR identification results from mixed samples are not directly comparable across laboratories without prior polymer-specific validation. In the wider context of environmental sample analysis, this highlights a persistent comparability issue between published datasets, not only across laboratories, but also within the same laboratory following changes to reference libraries or instrument software. This is particularly important when data are used to inform policy, define critical environmental thresholds, or where source apportionment is used to underpin the development of mitigation strategies targeting specific polymers (e.g. reducing LDPE residues of plastic mulch films in agricultural soils), as we have shown here that polymer identification is highly dependent on the laboratory-specific analytical configuration, including instrument, software, reference library and associated analytical workflows. This is further complicated by the fact that environmentally weathered polymers exhibit altered spectra compared to their pristine equivalents^12,21,22^, differences that are largely unaccounted for in current libraries. This is supported by the analysis of weathered polymer particles from soil matrices performed by four of the same laboratories in the present study, which showed a decline in median match quality of 3.4–45.1% points (*n* = 120 samples per country) relative to the pristine polymer median match reported here^33^ . This highlights a systematic misalignment of current polymer libraries and use for the identification of environmentally relevant microplastic particles.

### MP quantification is highly instrument-dependent despite standardised protocols

The high variability in PE recovery rates across all three matrices highlights that the use of standardised extraction protocols alone does not ensure a comparable quantification outcome. Whilst a higher variability for PE recovery rates from soil matrices was expected due to differing soil types and properties, the persistent variability for standardised matrices (bentonite clay, silicon dioxide) suggests that variability is associated with differences in laboratory-specific analytical configurations, including instrumentation, optical settings (e.g. excitation and emission wavelengths)^18,23^, and/or chemicals (i.e. Nile Red). The use of ImageJ for the particle analysis of fluorescent microscope images is widely accepted, and whilst this step can certainly introduce variability, the threshold settings in the Macros were optimised for each laboratory to mitigate this. Although the proportion of membrane analysed varied between laboratories (2–100%), particle counts were normalised to the total membrane area prior to recovery calculations. Furthermore, the laboratories analysing < 10% of the membrane independently verified that increasing the analysed area to 10% did not significantly alter particle counts, suggesting that membrane subsampling was unlikely to be a dominant source of the observed inter-laboratory variability, although it may have contributed to overall analytical uncertainty. The occurrence of recovery rates exceeding 100% (China 1) indicates a systematic overestimation of particles, whilst the majority of laboratories faced the opposite issue of underestimating particle counts (Fig. 6), suggesting systematic differences in particle detection efficiency between laboratories. Similar results were presented in a previous inter-laboratory study, showing a decrease in recovery rates with increasing matrix complexity and decreasing particle size^9,24^. These findings can also be considered in the context of the recently published ISO 16094-2:2025 guidance, which recommends recovery rates exceeding 60% for acceptable method performance (it is noteworthy that this standard evaluated IR methods in water-based matrices). Whilst several laboratories in the present study achieved or exceeded this benchmark for at least one matrix, the majority of recovery estimates fell below this threshold and substantial variability remained between laboratories despite the use of harmonised protocols. This highlights that utilising a harmonised approach alone does not necessarily ensure comparable analytical performance across laboratories and further emphasises the need for routine recovery validation and inter-laboratory quality assurance.

These findings complement the IR identification results, where laboratory-specific analytical configurations, including reference libraries and instrumentation, were associated with substantial inter-laboratory variability in match quality and accuracy. Combined, these results demonstrate that both polymer identification and quantification workflows are highly dependent on laboratory-specific analytical configurations, and that inter-laboratory comparability requires a more in-depth standardisation approach beyond procedural harmonisation. This is particularly timely, as the current consensus within the microplastic research community is that there is a definitive need for better standardisation to allow comparability of studies^6–8^, however we have shown here that protocol standardisation alone is insufficient.

An important limitation of the present study is that instrument type, software, reference library, spectral pre-processing, and operator were inherently linked within each participating laboratory. Consequently, although substantial inter-laboratory variability was observed, the relative contribution of each analytical component could not be isolated within the international comparison. The observed differences should therefore be interpreted as reflecting laboratory-specific analytical configurations rather than any individual methodological factor. The controlled UK library-modification case study partially addressed this limitation by isolating the influence of reference library composition on a single analytical platform, demonstrating that library composition alone can substantially influence identification accuracy. Future studies systematically varying individual analytical components would further improve understanding of their relative contributions to inter-laboratory variability.

## Conclusion & recommendations

In this inter-laboratory comparison study, we demonstrated that MP analysis is strongly influenced by laboratory-specific analytical configurations, including instrument, software, reference library, and associated analytical workflows, affecting both IR identification and fluorescent microscopy quantification workflows. We showed high variation in spectral match quality and accuracy for individual pristine polymer standards across laboratories, highlighting that match quality thresholds are not universally applicable. Moreover, these differences aligned with the variation observed in mixed polymer standards, indicating a systematic compositional bias associated with laboratory-specific analytical configurations. This was further supported by the UK case study, which demonstrated that modifying the reference library using representative spectra substantially altered identification performance under the analytical conditions examined. However, as the modified library was evaluated using the same polymer standards from which the additional spectra were derived, the magnitude of the observed improvement should be considered illustrative rather than an independent validation of library performance. In addition, we have shown that MP quantification via fluorescent microscopy resulted in recovery rates spanning < 20% to > 130% for standardised matrices despite the use of standardised extraction and analysis protocols, further highlighting the large influence of laboratory-specific analytical configurations on reported outcomes. In summary, our results emphasise that inter-laboratory comparability cannot be achieved by the use of standardised protocols alone, but requires at minimum thorough reporting of instrument types, reference libraries, and the use of shared reference materials for calibration to account for uncertainties associated with laboratory-specific analytical configuration.

Placing these findings into a wider environmental context raises important questions regarding the comparability, accuracy, and reliability of results reported in the current literature. The observed polymer-specific biases and inter-laboratory analytical differences in this study ultimately complicate environmental interpretations. Without transparent reporting and standardisation of reference systems, comparisons across regions, studies, and time are inherently confounded by analytical artefacts rather than true environmental variability. This is particularly relevant when scientific data are used to inform policy, legislation, and mitigation strategies. There is a clear, urgent need for harmonisation of approaches across laboratories, however, as we have shown here, this needs to extend beyond the standardisation of protocols and include a wider-reaching use of standard reference materials and harmonisation of reference libraries. Importantly, for environmental samples, the current state of manufacturer-supplied reference libraries is not fit for purpose and needs to include relevant environmentally weathered spectra (as pristine polymer spectra are insufficient proxies for environmental samples) and improved internal software-matching algorithms.

Although the present study demonstrates substantial inter-laboratory variability, it was not designed to isolate the relative contribution of instrument hardware, software, reference library, pre-processing workflow, and operator, which were inherently linked within each participating laboratory. Accordingly, the recommendations below focus on improving the overall analytical configuration rather than any single methodological component. Based on our findings, in addition to standardising protocols, our recommendations for IR polymer identification are to (i) report the instrument, manufacturer, and reference library, as well as any modifications made; (ii) avoid universal thresholds across instruments and instead validate laboratory-specific analytical performance with known standards prior to applying threshold criteria; (iii) validate match quality and accuracy of libraries using known polymer standards; (iv) report polymer-specific performance metrics; and (v) adopt inter-laboratory calibration standards to produce comparable baseline results that can be referred to when comparing studies.

Similarly, for fluorescent MP quantification, we recommend (i) the use of standardised recovery experiments from known matrices and the environmental matrix under investigation, to establish laboratory-specific correction factors; (ii) reporting of instrument type, manufacturer, imaging parameters, and excitation/emission wavelength configurations; and (iii) reporting of relevant metadata, including chemical specifications (e.g. Nile Red supplier, purity, and concentration) and membrane types.

However, as we have shown here, harmonisation at the laboratory level only addresses the issues of instrument and library effects to a degree. To make IR spectroscopy and fluorescent microscopy fit for purpose for the analysis of environmental MP samples, manufacturers play a crucial role. We therefore recommend that manufacturers (i) provide transparent, accessible library metadata, including library composition and source materials; (ii) provide confidence metrics beyond match quality; (iii) provide validation datasets (e.g. for accuracy) for individual polymers and mixed samples per instrument; (iv) provide calibration standards (e.g. to calibrate signal intensity prior to fluorescent analysis); and (v) ideally co-develop standardised reference libraries that can be used across instruments. Only through shared efforts between researchers and manufacturers can MP analysis of environmental samples become a comparable, reliable and effective monitoring tool.

## Materials and methods

### Participating laboratories and workflows

The following countries participated in this inter-laboratory study: Australia, China (one institution for IR spectroscopy, two institutions for MP quantification), Egypt, India, Sri Lanka, UK, and Vietnam. All standards were prepared in the UK and shipped to partnering countries in clean glass vials. A comprehensive overview of consumables and instruments used for MP quantification and identification is provided in the supplementary material (Table S1-S5). Briefly, IR spectroscopy instruments included an Agilent LDIR 8700, a Bruker Lumos II (µFTIR), a Bruker Alpha II (ATR-FTIR), a Perkin Elmer Spectrum 3 with Spotlight 200i (µFTIR), and two Thermo Fisher Scientific Nicolet iN10 MX systems (µ-ATR-FTIR), spanning four manufacturers and three instrument types. Reference libraries included manufacturer-specific libraries (Agilent Microplastic Starter 2.0, Bruker OPUS, and Thermo Fisher OMNIC Picta) as well as the open-source library Open Specy^5^. Fluorescent microscopes included instruments from five manufacturers (Zeiss, Leica, Olympus, Biobase, and Optika) with mercury, halogen, and LED light sources and Nile Red as the fluorescent dye. The use of plastic containers/implements was avoided for all analytical steps and substituted with metal or glass where possible. All countries followed standardised protocols, only differing in suppliers of chemicals/consumables and instrument types (Table S1-S5).

### Assessment of match quality and accuracy of known polymer standards via IR spectroscopy

The standards consisted of 9 individual polymer types (PET, PP, PTFE, PU, PC, PE, PS, PA, PVC) with different size ranges (Table S6). Each country identified 10 randomly selected particles per standard. The analysis was carried out blind, i.e. participating countries (apart from UK) were unaware of what polymer types were included in each standard vial. Sri Lanka had no capability to analyse polymer standards via IR spectroscopy and was therefore excluded from this analysis. India was unable to analyse ‘standard 3’ (PTFE) due to sample loss. As true polymer identities were known for all standards, identifications could be classed as correct or incorrect. The assessed metrics included (i) the median spectral match quality per country, (ii) the overall identification accuracy, and (iii) polymer-specific accuracy and spectral match quality.

### Effect of library modification on match quality and accuracy: UK case study

To assess the influence of reference library composition on IR identification performance, the UK standard dataset (*n* = 90 particles; 9 polymer standards x 10 particles each) was re-analysed using an updated reference spectral library on the same instrument (LDIR 8700; Agilent Technologies, USA). To update the original library which was provided by the manufacturer (Library 1), spectra from the known polymer standard particles were imported to the library, then the standard analysis was repeated using the modified library (Library 2) for the same particles. For each particle, the best library match and corresponding match quality (%) were recorded. True polymer identities were known for all standards, enabling classification of identifications as correct or incorrect. The assessed metrics included (i) the median match quality per spectral library, (ii) the overall identification accuracy, (iii) polymer-specific changes in accuracy and match quality, and (iv) particle-level paired differences in match quality.

### Distribution patterns of polymer types in mixes

In addition to individual standards, each laboratory received one homogenised polymer mixture in triplicate form, containing the same nine polymers in known weight proportions (Table S7). Each laboratory analysed all three replicates of the mixed polymer standard (from here on referred to as Mix 1–3), selecting 50 random particles per replicate. India was unable to analyse Mix 1 and 2 due to sample loss, therefore only Mix 3 is included. Consequently, within-laboratory repeatability could not be assessed for India, although its remaining replicate was retained for the inter-laboratory comparison of polymer composition. Because the true polymer identity of individual particles in the mix was unknown, focus was placed on distributional patterns rather than particle-level correctness. The assessed metrics included (i) the relative polymer identification frequencies per country and replicate, (ii) consistency within laboratories, (iii) inter-laboratory compositional similarity, and (iv) potential effects of colour, size, and shape. Polymer identifications were normalised to proportions within each replicate to allow inter-laboratory comparison.

### Quantification of MPs in solid matrices using fluorescent microscopy and ImageJ

In addition to Australia, China, Egypt, India, UK, and Vietnam, standards for the assessment of particle quantification were also analysed in Sri Lanka and a second institution in China (China 2). The standards consisted of three matrices: silicon dioxide (SD; quartz sand), bentonite clay (BT), and country-representative soil; all with and without 0.04% (*w*/*w*) polyethylene (PE; 34–50 μm particle size). See Table S5 for material specifications. SD and BT were selected to represent two commonly found soil textures (sand, clay), and soil was selected representative of each country’s prevalent soil types. The soil was collected by each laboratory and spiked with PE (provided by UK) to 0.04% (*w*/*w*). The UK also determined the number of PE particles in the mass required to achieve the 0.04% (*w*/*w*) concentration in 5 g matrix to estimate the average particle number per sample and therefore estimate maximum particle number recoverable. For the calculation of recovery rates all samples were blank corrected.

All standards were analysed in triplicates alongside blanks (no matrix, only reagents), following a standardised extraction and analysis protocol^20^. The details of reagents, consumables, and instruments used in each country can be found in the supplementary material (Table S1-S5). Briefly, supplied samples were weighed out to 5 ± 0.05 g (dry weight) into a clean glass beaker. For density separation, 230 ml saturated NaCl were added to the beaker, the solution was homogenised and left standing overnight. Following this, the supernatant was decanted into a new glass beaker, and 4 M NaOH was added to a fixed volume of 500 ml, after which the solution was homogenised and left standing overnight for digestion of organic material. The supernatant was then decanted into a new glass beaker and 250 µl of Nile Red solution was added, homogenised, and incubated in the dark at room temperature for 30 min before vacuum filtration onto a suitable membrane (e.g., polycarbonate, mixed cellulose ester, nylon, cellulose). Beakers were covered with aluminium foil for all steps to minimise laboratory contamination.

Particle quantification on membranes was carried out using fluorescent microscopy within each country. A total of 2–100% of the membrane area were analysed, depending on country (Table S3), and resulting images were processed using ImageJ (v. 1.54f)^25^. Briefly, a Macro was customised for each country, scaling images accordingly, applying a suitable colour threshold with the default thresholding method, adjusting the brightness level to a suitable setting (according to the average best fit of 10 randomly selected images), and exporting particles’ Feret’s diameter. Data were filtered to only include particles with Feret’s diameter of 20–500 μm and scaled to equivalent particle number of 100% membrane area (= 5 g dry weight)^20^. Total particle number was then divided by 5 to express particles g^− 1^ (dry weight).

### Statistics and visualisation

All analyses were conducted using R (v. 4.3.3)^26^ with packages ‘*lme4*’ (v. 1.1–35.1)^27^, ‘*ggplot2*’ (v. 3.5.0)^28^, ‘*ggeffects*’ (v. 2.3.2)^29^, ‘*patchwork*’ (v. 1.2.0)^30^, ‘*tidyverse*’ (v. 2.0.0)^31^, and ‘*janitor*’ (v. 2.2.1)^32^.

For the pure polymer standards, identification outcome was coded as a binary response (correct/incorrect) based on the known true polymer identity of each standard particle. To quantify polymer-specific effects while accounting for inter-laboratory differences, a generalised linear mixed-effects model (GLMM) with a binomial error distribution and logit link was fitted. Correctness was modelled as a function of polymer identity with country included as a random intercept to account for lab-level differences in baseline performance. Polymer reference levels were adjusted (using PET as reference) to support interpretable effect sizes. The model was extended by adding the standardised match quality score as a fixed effect to test whether higher match quality increased the probability of correct identification. To reduce instability arising from sparse polymer-specific errors and to reflect empirically observed differences in difficulty, polymers were grouped into three categories: easy (PTFE, PS, PVC), moderate (PET, PC, PA, PE), and difficult (PP, PU). A final GLMM was fitted with polymer difficulty group and standardised match quality as fixed effects and country as a random intercept. Model outputs were used to estimate adjusted probabilities of correct identification across the observed range of match quality, applying bias correction for population-level predictions.

The effect of spectral library modification on match quality changes was evaluated using a paired Wilcoxon signed-ranked test.

## Supplementary Information

Below is the link to the electronic supplementary material.

Supplementary Material 1

## Data Availability

All data has been deposited with the EIDC and is openly available here: https://doi.org/10.5285/c0407da7-acd0-4f5f-a1f3-5516206ba787.

## Abbreviations

EBS: Ethylene bis-stearamide
PA: Polyamide
PAAU: Poly(aromatic aliphatic urethane)
PC: Polycarbonate
PE: Polyethylene
PET: Polyethylene terephthalate
PHBV: Poly(3-hydroxybutyrate-*co*-3-hydroxyvalerate)
PP: Polypropylene
PS: Polystyrene
PTFE: Polytetrafluoroethylene
PU: Polyurethane
PVC: Polyvinyl chloride
