# Supplementary material for: Instrument-dependent variability affects both microplastic polymer identification and quantification: an inter-laboratory comparison using polymer standards
Source: Sci Rep. 2026 Jul 28;16:34427. doi: 10.1038/s41598-026-64477-w (PMC13710154; doi:10.1038/s41598-026-64477-w)
Supplement: Supplementary file 1 [file 41598_2026_64477_MOESM1_ESM.pdf]

Supplementary material

Instrument-dependent variability affects both microplastic polymer identification and quantification: An inter-laboratory comparison using polymer standards

Martine Graf[1*], David R. Chadwick[1], Yingming Sun[2], Fan Ding[2], Siyang Ren[1,3], Kai Wang[3], Changrong Yan[4], Shitong Li[2], Xuejun Liu[3], Mona Tolba[5,6], Ahmed Mosa[5], Sanjay Singh[7], Tapan Adhikari[7], Asit Mandal[7], Jyoti K. Thakur[7], Ajay Yadav[7], Meththa Gimhani[1,8], Achini Dias[1,8], Surani Chathurika[8,9], Mojith Ariyaratne[10], Hao T. T. Dang[11], Hien V. Nguyen[11], Tien T. Minh[11], Samantha Viljoen[12], Davey L. Jones[1]

[1] School of Environmental & Natural Sciences, Bangor University, Bangor, UK
[2] College of Land and Environment, Shenyang Agricultural University, Shenyang, China
[3] State Key Laboratory of Nutrient Use and Management, College of Resources and Environmental Sciences; National Academy of Agriculture Green Development; Key Laboratory of Plant-Soil Interactions of Ministry of Education, National Observation and Research Station of Agriculture Green Development (Quzhou, Hebei), China Agricultural University, Beijing, China
[4] Institute of Environment and Sustainable Development in Agriculture, Chinese Academy of Agricultural Sciences, Beijing, China
[5] Soils Department, Faculty of Agriculture, Mansoura University, Mansoura, Egypt
[6] Plant Nutrition Department, National Research Centre, Giza, Egypt
[7] ICAR – Indian Institute of Soil Science, Bhopal, India
[8] Sri Lanka Institute of Nanotechnology, Homogama, Sri Lanka
[9] Department of Urban Bioresources, Faculty of Urban and Aquatic Bioresources, University of Sri Jayawardenepura, Nugegoda, Sri Lanka
[10] Department of Crop Science, Faculty of Agriculture, University of Peradeniya, Peradeniya, Sri Lanka
[11] Soils and Fertilizers Institute, Ha Noi, Vietnam
[12] Bioplastic Innovation Hub, Food Futures Institute, Murdoch University, Perth, Australia

Corresponding author:	* Martine Graf
Corresponding author address:	School of Environmental & Natural Sciences, Bangor University, Bangor, Gwynedd, LL57 2UW, UK
Corresponding author e-mail:	m.graf@bangor.ac.uk
ORCID:	0000-0001-7994-0529

Table S1. Metadata for infrared polymer standard analysis.

Country	Instrument manufacturer	Instrument type	Instrument model	Place of analysis	Spectra reference library
Australia	Thermo Fisher Scientific	μ-ATR-FTIR	Nicolet iN10 MX	Curtin University (Australia)	Instrument library (incorporated polymer-specific library/libaries in OMNIC Picta software)
China 1	Bruker	μ-FTIR	Lumos II	Chinese Academy of Agricultural Sciences (China)	Instrument library (incorporated polymer-specific library/libaries in OPUS software)
Egypt	Bruker	ATR-FTIR	Alpha II	Mansoura University (Egypt)	Open Specy
India	Perkin Elmer	μ-FTIR	Spectrum 3 with Spotlight 200i	CIPET (India)	Open Specy
UK	Agilent Technologies	Quantum cascade laser direct infrared system	LDIR 8700	Bangor University (UK)	Instrument library (Agilent Microplastic Starter 2.0 modified with standard polymer spectra)
Vietnam	Thermo Fisher Scientific	μ-ATR-FTIR	Nicolet iN10 MX	Hanoi University of Science and Technology (Vietnam)	Instrument library (incorporated polymer-specific library/libaries in OMNIC Picta software)

Table S2. Metadata for fluorescent microscopes and filtration systems.

**Country**	**Filtration system**			**Fluorescent microscope**				
	Manufacturer: Filtration system	Manufacturer: Vacuum pump	Vacuum pump type	Microscope manufacturer	Microscope model	Excitation wavelength (nm)	Emission wavelength (nm)	Place of analysis
Australia	Pyrex	Javac	IP55 AS1359	Zeiss	Axioscan 7 (with LED light source)	553	568	University of Western Australia (Australia)
China 1	Tianjin Jinteng Experiment Equipment Co.	Tianjin Jinteng Experiment Equipment Co.	SHZ-DIII	Leica Microsystems	FIM (with 100 W mercury light source)	340-360	425	Shenyang Agricultural University (China)
China 2	Tianjin Jinteng Experiment Equipment Co.	LiChen Scientific	LC-85L	Olympus	BX53 (with 120 W mercury light source)	530-550	575	China Agricultural University (China)
Egypt	Pyrex	Rocker	Rocker 300	Leica Microsystems	DM2500 (with halogen light source)	450-490	515	National Research Centre (Egypt)
India	General machine tools	Prominent	PS2-800	Optika Microscopes	B-353LD2 (with LED light source)	470	495	ICAR-IISS (India)
Sri Lanka	Glassco	Value	2FY-1B	Olypmus	BX61 (with 100 W halogen light source)	475	530	Sri Lanka Institute of Nanotechnology (Sri Lanka)
UK	Pyrex	Vacuubrand	ME 8C NT +2AK	Zeiss	Axioplan 2 (with 100 W mercury light source)	470	495	Bangor University (UK)
Vietnam	Tianjin Jinteng Experiment Equipment Co.	Gast	DOA-P504-DN	Biobase	XY-2 (with LED light source)	460-490	515	Vietnam Academy of Science and Technology (Vietnam)

Table S3. Metadata for filter membranes.

Country	Membrane manufacturer	Membrane type	Membrane product number	Membrane pore size (μm)	Membrane diameter (mm)	Real membrane diameter (mm) †	Real membrane area (mm^2) ‡	Image size taken for analysis (mm x mm)	Membrane area analysed (%)
Australia	Labquip	Nylon	N14722	0.22	47	37	1075.21	2.07 x 2.87	100
China 1	Tianjin Jinteng Experiment Equipment Co.	MCE (mixed cellulose ester)	365642	0.20	50	40	1256.00	2.49 x 1.87	5
China 2	Tianjin Jinteng Experiment Equipment Co.	MCE (mixed cellulose ester)	365643	1.20	50	40	1256.00	1.80 x 1.30	2
Egypt	Chromtech	Nylon	CTF-NY020-47	0.22	47	40	1256.64	6.17 x 4.63	22.74
India	Whatman	Cellulose	1442-047	2.50	47	40	1256.64	3.64 x 3.48	10.09
Sri Lanka	Labfil	Nylon	C0000303	0.22	47	35	1164.71	3.57 x 4.74	14.50
UK	Sterlitech	Polycarbonate (PCTE), PVP-free	PCTF0247100	0.20	47	38	1134.11	3.70 x 4.92	16.05
Vietnam	Alain Lab	Nylon	MFNY047045	0.45	47	40	1256.64	1.70 x 0.96	100

† The diameter of the membrane area that contains particles after filtration. ‡ Calculated using the real membrane diameter.

Table S4. Metadata of soil standards and chemicals.

Country	Soil standards			Chemicals						
	Soil dry weight (g)	Soil class	Soil texture	Nile Red supplier	Nile Red † product number	Nile Red purity (%)	NaOH ‡ supplier	NaOH product number	NaCl § supplier	NaCl product number
Australia	5 ± 0.05	Arenosol	Sand	Sigma Aldrich	72485	99	Row Scientific	CK150515	Chem-Supply	9326410006496
China 1	5 ± 0.05	Cambisol	Loam	Macklin	N815046	95	Heng Xing	30225	Heng Xing	30144
China 2	5 ± 0.05	Umbrisol	Loam	Yuanye Bio-Technology Co.	S19279	98	Sinopharm Chemical Reagent	UN No.1823	Sinopharm Chemical Reagent	10019318
Egypt	5 ± 0.05	Arenosol	Sand	Thermo Fisher Scientific	10464311	99	Alpha Chemika	AL4159	Alpha Chemika	AL4105
India	5 ± 0.05	Vertisol	Clay	Sigma Aldrich	72485	99	Sigma Aldrich	n/a	Sigma Aldrich	n/a
Sri Lanka	2.5 ± 0.05	Luvisol	Sandy-glay-loam	MP Biomedicals	215174490	99	Chem-Lab	CL00.1492	Loba Chemie Pvt. Ltd.	5820
UK	5 ± 0.05	Cambisol	Clay-loam	BLD Pharmatech Ltd.	BD306389	98	Sigma Aldrich	221465	Sigma Aldrich	S9888
Vietnam	0.5 ± 0.05	Acrisol	Sandy-silt-loam	Macklin	N815046	95	Xi-Long	n/a	Xi-Long	n/a

† Nile Red CAS number: 7385-67-3; ‡ NaOH CAS number: 1310-73-2; § NaCl CAS number: 7647-14-5.

Table S5. Metadata for standard materials used for recovery tests.

Material	Supplier	Product number	CAS number
Polyethylene	Sigma Aldrich	434272	9002-88-4
Silicon dioxide	Sigma Aldrich	18649	60676-86-0
Bentonite clay	Sigma Aldrich	285234	1302-78-9

Table S6. Material specifications of individual polymer standards analysed via IR spectroscopy in each country. Average and median particle sizes were determined by the UK using the LDIR 8700 chemical imaging system's reported diameter (n = 10).

Standard #	Polymer type	Supplier	CAS	Product number	Measured average particle size ± SD (µm)	Measured median particle size (µm)	Particle type and shape	Colour	Weight per vial (mg)
1	PET	Goodfellow	25038-59-9	ES30-PD-000132	130 ± 112	97	Powder - circular	White	1
2	PP	Parchvale Ltd.	9003-07-0	Glissawax 14205	13 ± 23	5	Powder – circular	White	0.5
3	PTFE	Goodfellow	9002-84-0	FP30-FB-000122	121 ± 266	12	Fibre – elongated	Brown	0.5
4	PU	Goodfellow	9009-54-5	UR30-GL-000101	2150 ± 948	2160	Granule – fragment	Clear	20
5	PC	Goodfellow	25766-59-0	CT30-GL-000111	261 ± 469	38.5	Granule – fragment	Red	2
6	PE	Sigma Aldrich	9002-88-4	434272	22 ± 37	8	Powder – circular	White	0.5
7	PS	Sigma Aldrich	9003-53-6	441147	764 ± 968	88	Granule – fragment	Clear	2
8	PA	Goodfellow	25038-54-5	AM30-PD-000155	31 ± 42	14	Powder – circular	White	0.5
9	PVC	Sigma Aldrich	9002-86-2	189588	194 ± 107	224	Powder - circular	White	1

Table S7. Composition of polymer mix standard (analysed in triplicates: Mix 1-3) analysed via IR spectroscopy in each country.

**Polymer type**	**Weight added (mg)**	**Equivalent of total weight (% *w/w*)**
PET	0.5	2.04
PP	0.15	0.61
PTFE	0.25	1.02
PU	20	81.80
PC	1.25	5.11
PE	0.15	0.61
PS	1.5	6.13
PA	0.15	0.61
PVC	0.5	2.04

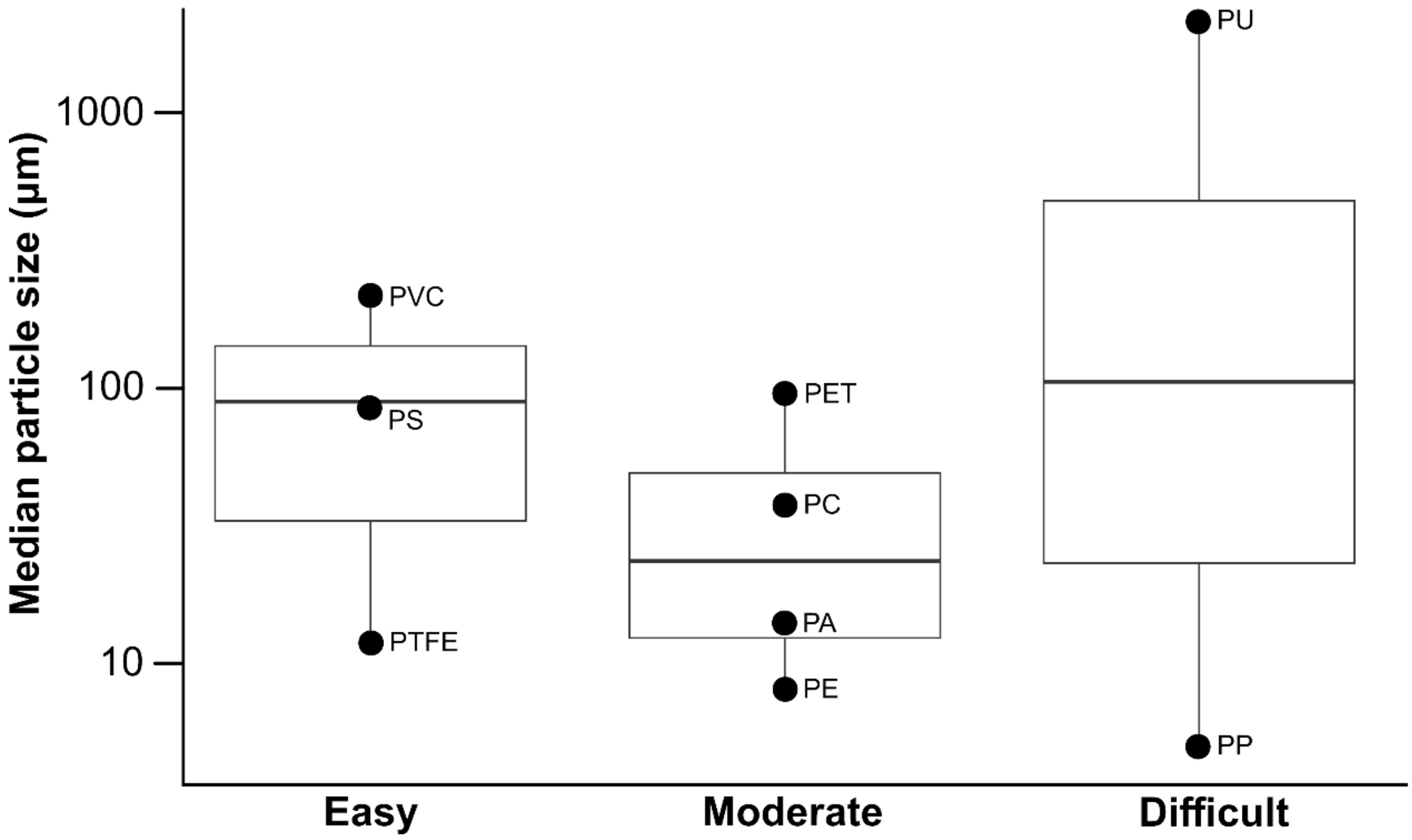

Figure S1. Median particle size distribution by polymer difficulty group. Boxplots show the distribution of median particle size (µm, log scale) for polymers grouped by identification difficulty (easy, moderate, difficult). Points represent individual polymer types within each group.

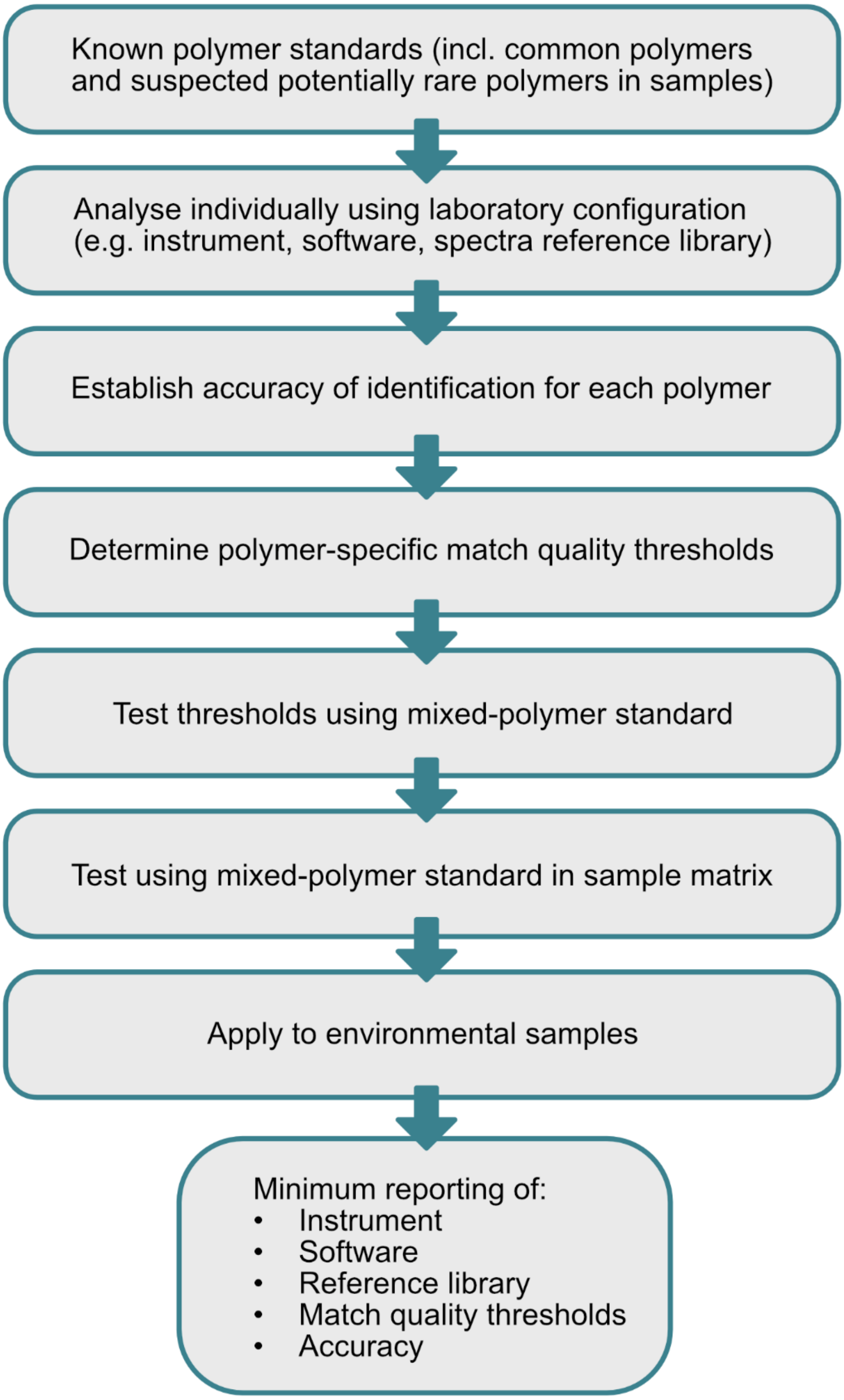

Figure S2. Framework for the determination of polymer-specific match quality thresholds for implementation in laboratory QA/QC procedures regarding polymer identification using IR spectroscopy.
